# Population genetics of 30 insertion/deletion polymorphisms in the Kuwaiti population

**DOI:** 10.1007/s00414-019-02180-4

**Published:** 2019-11-14

**Authors:** Mahdi Haidar, Hussain Alsaleh, Penelope R. Haddrill

**Affiliations:** 1 Centre for Forensic Science, Department of Pure and Applied Chemistry, University of Strathclyde, Glasgow, Scotland, UK; 2 Kuwait Identification DNA Laboratory (KIDL), General Department of Criminal Evidence, Ministry of Interior, Kuwait City, Kuwait

**Keywords:** DIPplex, Kuwait, Forensic genetics, INDEL, Population genetics

## Abstract

This study evaluates the forensic utility of the 30 insertion and deletion (indel) markers contained in the Qiagen Investigator® DIPplex kit in the Kuwaiti population (*n* = 150). All but one of the 30 markers were shown to conform to the expectations of the Hardy-Weinberg Equilibrium. Linkage disequilibrium tests showed no statistically significant deviation from independence. The high combined power of discrimination (CPD > 99.999%) and low combined match probability (CMP) of 2.736 × 10^−13^ provide a satisfactory level of discrimination, allowing the DIPplex loci to be used as forensic markers for individual identification in Kuwait. The paternity indices indicate the usefulness of the DIPplex kit as a supplementary typing system for challenging paternity cases in Kuwait.

**Electronic supplementary material:**

The online version of this article (10.1007/s00414-019-02180-4) contains supplementary material, which is available to authorized users.

Indel (insertion and deletion) markers are a class of variants that have relatively recently been developed for use as genetic markers, and which have been increasing in use in the forensic community. These polymorphisms are useful because they combine many of the benefits of SNP markers but use simple STR typing techniques, making it feasible to implement them in forensic genetic laboratories [1–4]. A commercial kit, the Investigator® DIPplex (Qiagen, Hilden, Germany), includes 30 indel markers and the Amelogenin sex chromosome marker in a single PCR reaction. To date, no study has investigated the DIPplex markers in the Kuwaiti population; therefore, this study aims to evaluate the forensic utility of these markers in this population, as well as to increase the amount of population genetic data available for this under-represented population.

A total of 150 blood samples were collected from unrelated Kuwaiti individuals; all donors first signed a consent form, in accordance with ethical approval for the study, granted by the Department of Pure and Applied Chemistry Ethics Committee at the University of Strathclyde. DNA was extracted using the AutoMate Express™ with the PrepFiler Express™ Kit (Thermo Fisher Scientific, USA), and quantified using the Promega PowerQuant™ System (Madison, WI, USA), performed on a 7500 Real-Time PCR instrument (Thermo Fisher Scientific). Sample concentrations were standardised according to the manufacturer’s recommendations (0.5 ng/μL). Amplification was carried out using the GeneAmp PCR System 9700 (Thermo Fisher Scientific). DNA fragment analysis was performed on a 3500 Genetic Analyzer (Thermo Fisher Scientific). Genotype data were analysed using *GeneMapper® ID-X* software version 1.4 (Thermo Fisher Scientific). The Analysis of Molecular Variance (AMOVA) statistical test in *Arlequin* version 3.5 was used to detect genetic structure among and within individuals, and to calculate *F*-statistics [5]. *Arlequin* was used to calculate allele frequencies, observed and expected genotype heterozygosity and to test for Hardy-Weinberg Equilibrium (HWE). *Genepop* version 4.7 was used to test linkage disequilibrium (LD) [6]. Forensic parameters were calculated using *Promega PowerStats* version 1.2.

Population statistical parameters for the 150 Kuwaiti samples genotyped are shown in Supplementary Table 1. The AMOVA test showed that 96% of genetic variation in the Kuwaiti sample was found within individuals and only 4% among them. The average inbreeding coefficient (*F*_*IS*_) between individuals was 0.0435, indicating the presence of mild genetic inbreeding in the Kuwait population. Comparing this *F*_*IS*_ value with those found in the literature for the Kuwaiti population, this is somewhat higher. For instance, the average inbreeding coefficient calculated by Alsmadi at al. (2013) based on genome-wide SNP markers was 0.0196. The high *F*_*IS*_ value for the DIPplex markers can be attributed to their low number (30 markers) compared with the number of markers that other papers have used (mostly genome-wide markers), and the sample size used in this study [7]. Heterozygosity ranged from 0.333 to 0.560 (mean = 0.461), indicating a high-level of diversity of the markers across the Kuwaiti population. Three markers (HLD56, HLD88 and HLD97) violated the expectations of the Hardy-Weinberg Equilibrium (HWE) at a significance level of 0.05. When the Bonferroni correction (calculated by dividing the significance level of 0.05 by the number of tests, i.e. 0.05/30 = 0.0016) was applied, HLD56 and HLD97 did not statistically deviate from the expectations of HWE (*P* values all > 0.0016). However, significant departure from HWE was still observed in HLD88. The departure was a result of an excess of homozygotes, which could be due to either allele drop out or inbreeding; the latter is supported by a mild level of inbreeding within the Kuwaiti population. From the 435 tests of all possible pairwise combinations of indel markers, 21 pairs showed significant linkage disequilibrium (*P* value < 0.05). However, after Bonferroni correction (calculated by dividing the significance level of 0.05 by the number of tests, i.e. 0.05/435 = 0.0001), no pairs of markers showed significant linkage disequilibrium (*P* values all > 0.0001). Therefore, the DIPplex loci are statistically independent and can be used to calculate match probabilities in the Kuwaiti population.

Forensic statistical parameters for the 150 Kuwaiti samples genotyped are shown in Supplementary Table 1. The combined match probability (CMP) of the 30 markers was 2.736 × 10^−13^ in the Kuwaiti population, meaning that the probability of finding identical indel profiles in two randomly selected individuals is one in 3.70 × 10^12^. The combined power of discrimination (CPD) of the markers was greater than 99.999%. These findings indicate that the DIPplex markers are highly discriminatory for human identification purposes in this population. The combined power of exclusion (CPE) for the 30 indels was calculated as 99.46% and their typical paternity index (TPI) values ranged between 0.750 and 1.136 (average = 0.936) indicating that indel markers are not informative for paternity testing as a stand-alone system in the Kuwaiti population. However, the advantage of their low mutational rate and the simple techniques required to genotype them means they may be useful for investigating difficult paternity cases, especially when STR markers are inconclusive (for example if mutations exist in families) or in cases where extra markers are needed as supporting evidence. In general, this study confirms previous findings that these di-allelic indel markers may be useful for forensic purposes but they are less informative than STR markers, so more markers are required to achieve similar levels of power based on indels compared to STRs.

## Electronic supplementary material

ESM 1 (XLSX 16 kb)
